# Effects of different arachidonic acid supplementation on psychomotor development in very preterm infants; a randomized controlled trial

**DOI:** 10.1186/s12937-015-0091-3

**Published:** 2015-09-30

**Authors:** Ayham Alshweki, Alejandro Pérez Muñuzuri, Ana M. Baña, Ma. José de Castro, Fernando Andrade, Luís Aldamiz-Echevarría, Miguel Sáenz de Pipaón, José M. Fraga, María L. Couce

**Affiliations:** 1 Neonatology Unit, Department of Paediatrics, University Clinical Hospital of Santiago de Compostela. IDIS, CIBERER, Travesia Choupana, 15706, Santiago de Compostela, Spain; 2 Motabolism Group, BioCruces Health Research Institute, CIBER of Rare Diseases (CIBERER), Plaza de Cruces 12, 48903 Baracaldo, Vizcaya, Spain; 3 Neonatology Unit, Department of Paediatrics, La Paz University Clinical Hospital, P de la Castellana 261, 28064 Madrid, Spain

**Keywords:** Lipids, Nutrition, Arachidonic acid, Omega-6/Omega-3 Ratio, Preterm infants, Psychomotor development

## Abstract

**Background & aims:**

Nutritional supplementation with polyunsaturated fatty acids is important in preterm infants neurodevelopment, but it is not known if the omega-6/omega-3 ratio affects this process. This study was designed to determine the effects of a balanced contribution of arachidonic acid in very preterm newborns fed with formula milk.

**Methods:**

This was a randomized trial, in which newborns <1500 g and/or <32 weeks gestational age were assigned to one of two groups, based on the milk formula they would receive during the first year of life. Initially, 60 newborns entered the study, but ultimately, group A was composed of 24 newborns, who were given formula milk with an ω-6/ω-3 ratio of 2/1, and Group B was composed of 21 newborns, given formula milk with an ω-6/ω-3 ratio of 1/1. The infants were followed up for two years: growth, visual-evoked potentials, brainstem auditory-evoked potentials, and plasma fatty acids were periodically measured, and psychomotor development was assessed using the Brunet Lézine scale at 24 months corrected age. A control group, for comparison of Brunet Lézine score, was made up of 25 newborns from the SEN1500 project, who were fed exclusively with breast milk.

**Results:**

At 12 months, arachidonic acid values were significantly higher in group A than in group B (6.95 ± 1.55 % *vs.* 4.55 ± 0.78 %), as were polyunsaturated fatty acids (41.02 ± 2.09 % *vs.* 38.08 ± 2.32 %) achieved a higher average. Group A achieved a higher average Brunet Lézine score at 24 months than group B (99.9 ± 9 *vs.* 90.8 ± 11, *p* =0.028). The Brunet Lézine results from group A were compared with the control group results, with very similar scores registered between the two groups (99.9 ± 9 *vs.* 100.5 ± 7). There were no significant differences in growth or evoked potentials between the two formula groups.

**Conclusions:**

Very preterm infants who received formula with an ω-6/ω-3 ratio of 2/1 had higher blood levels of essential fatty acids during the first year of life, and better psychomotor development, compared with very preterm newborns who consumed formula with an ω-6/ω-3 of 1/1. Therefore, formula milk with an arachidonic acid quantity double that of docosahexaenoic acid should be considered for feeding very preterm infants.

**Trial registration:**

ClinicalTrials.gov Identifier NCT02503020.

## Background

Long-chain polyunsaturated fatty acids (LCPUFAs), arachidonic acid (AA omega-6; 20:4ω-6), and docosahexaenoic acid (DHA omega-3; 22:6ω-3), are required for the formation of non-myelinated cell membranes in the central nervous system, including in the retina [1, 2], hence their great importance in appropriate visual and cognitive development. LCPUFAs are transferred from mother to foetus mainly during the last trimester of pregnancy [2, 3]. At that time, and in the early neonatal period, sufficient levels of DHA and AA are required for the rapid synthesis of brain tissue, cellular differentiation, and active synaptogenesis [4, 5]. In premature infants, this crucial supply is interrupted and they exclusively depend on breast milk and other exogenous sources [5]. Moreover, very-low-birth-weight (VLBW) and very preterm infants are particularly vulnerable to LCPUFAs deficiency, given the virtual absence of adipose tissue at birth, the potential immaturity of fatty acid elongation/desaturation pathways, and inadequate fatty acid intake from formula milk if not breast fed [6].

Arachidonic acid is the most abundant ω-6 LCPUFA, and DHA is the most biologically important ω-3 LCPUFA in breast milk [7]. The ratio of AA to DHA in human milk is usually 1.5-2/1, but the variability is high, and the ratio is primarily determined by the habitual diet of the region or country [8, 9]. The nutritional requirements for LCPUFAs in preterm newborns are not clearly established, because optimal LCPUFA blood levels and accretion rate are not well known [10, 11].

It has been reported that newborns fed with DHA- and AA-supplemented formula had higher Bayley mental and psychomotor development scores, with no increase in morbidity or adverse events [12]. Likewise, other studies in term and preterm infants considered formulas containing DHA to be more appropriate, because of the frequent deficiency of DHA in the first days of life in VLBW infants [13, 14]. However, it must be remembered that AA is also a key component of cell membranes and is one of the most abundant fatty acids in the brain: it helps maintain hippocampal cell membrane fluidity [15], protects the brain from oxidative stress by activating peroxisome proliferator-activated receptor gamma [16], and activates syntaxin-3 (STX-3), a protein involved in the growth and repair of neurons [17]. In one study, term infants who were given supplemental AA showed significant improvements in intelligence, as measured with the Mental Development Index [18]. The dietary balance between ω-6 and ω-3 and their LCPUFA metabolites is likely to be important in humans of all ages, but perhaps even more so in preterm infants [19, 20].

Almost all preterm infant formulas contain AA supplementation. However, the formulas often contain an ω-6 to ω-3 ratio of 1/1. This study aimed to clarify the importance of higher formula AA values and establish the preferred omega-6/omega-3 ratio for supplementation of breast milk, by assessing anthropometric, visual, auditory, and psychomotor development in very preterm infants receiving diets supplemented with different AA/DHA ratios (1/1 *vs.* 2/1).

## Patients and methods

### Study design

This prospective randomized controlled double-blind trial was conducted to study nutritional supplementation in preterm infants <1500 g and/or between 25–32 weeks gestational age (GA) who were born at the University Clinical Hospital of Santiago de Compostela (CHUS). The infants were enrolled for a period of 14 months (from July 2011 to August 2012) and followed up from birth until 2 years of age. Milk formulas were provided either as adjunct to insufficient breast milk quantity or as full formula feeding. Breastfeeding was actively encouraged. Patients were randomly assigned to one of the two formula groups, according to the type of formula they were to receive. The group A formula was supplemented with AA and DHA with an ω-6/ω-3 ratio of 2/1. The group B formula was supplemented with AA and DHA with an ω-6/ω-3 ratio of 1/1. The primary outcome was psychomotor development, assessed with the Brunet Lézine scale at 2 years of age (Early Care Unit, CHUS). The secondary outcomes were plasma levels of fatty acids at 3 months, 6 months, and 12 months (Metabolic Unit, Cruces Hospital, Bilbao); visual- and auditory-evoked potentials at 6 and 12 months of age (Neurophysiology Unit, CHUS); and anthropometric measurements (weight, length, and head circumference) at 3, 6, 9, 12, 18, and 24 months of age (Neonatology Unit CHUS).

The Brunet Lézine assessment results from the 2 sample groups were compared with the results from 25 preterm infants (<1500 g) from the same hospital (CHUS) who were fed exclusively with human milk. Information was used from the <1500 g preterm data registry of the Spanish Society of Neonatology (SEN 1500). This program includes 62 hospitals and centres throughout Spain, CHUS being one of them. The 25 most recently-born preterm infants (<1500 g) who were exclusively breast-fed were chosen. There was no human milk bank available, therefore all infants were fed with their own mother's milk.

### Power calculation and randomization

The primary outcome of the study was psychomotor development. Differences in the Brunet Lezine score were expected; therefore, the sample size was calculated on this parameter using Brunet Lezine score results from the SEN 1500 study over 9 years (from 2003 to 2011). The absolute effect size was estimated for comparison between formula- and human milk-fed children: it was 7.2 points. The sample size was 30 infants in each group, achieving an observed power of 80 %. The double-blind randomized allocation of infants to a study formula was stratified for gender, and a block size of four was applied. The researcher that generated the random allocation sequence was not the same researcher that enrolled participants and assigned participants to interventions. Participants and investigators were blinded to formula allocation until all data analysis had been performed.

### Study population

During the data collection period, 3357 infants were born in our Hospital, of which 61 weighed <1500 g and/or were 25–32 weeks GA. One child was excluded during the first week due to a severe malformation; all other parents gave consent to participate in the study (30 infants in each group). Patients in the following situations were excluded: preterm infants with severe malformations, preterm infants with severe intraventricular haemorrhage or periventricular leukomalacia (more than grade 2), and neonates who did not need supplementary milk nutrition, *i.e.* breast-fed only children.

Sixty preterm infants entered the study in the first week of age. One died at 2 months, and communication was lost with two more: one at 3 months and the other at 6 months of age. Three preterm infants were excluded during their first two months because of severe intraventricular haemorrhage, and nine children converted to exclusive breastfeeding during the first six months. From 6 months until the end of the study, 45 children were included and followed up: 24 infants in group A and 21 in group B.

### Ethical statement

The study was approved by the Galician Research Ethics Committee, Spain. Written informed consent was obtained from all parents, after the experimental protocol had been explained to them in detail. The study was registered at ClinicalTrials.gov (NCT02503020).

### Dietary intervention

Breastfeeding was encouraged in all preterm infants. Neonates who could not meet more than half their milk requirements from their mothers’ milk alone at the end of the first week of age were included in the study. Infants in group A received milk formula for preterm infants containing a fixed amount of ω-3 (DHA) lipids (0.33 %) with an ω-6/ω-3 ratio of 2/1. Infants in group B received formula for preterm infants containing a similar quantity and quality of proteins, carbohydrates, lipids, vitamins, and micronutrients as group A, and a fixed amount of ω-3 lipids (0.37 %) but with an ω-6/ω-3 ratio of 1/1. It should be noted that neither of the formulas used contained other sources of ω-6 and ω-3 besides AA and DHA, therefore when this study refers to the ω-6/ω-3 ratio, it is equivalent to the AA/DHA ratio. Whilst in hospital, the two groups received similar quantities of fluids and calories (almost 160 ml/kg/day of fluids, and 120 kcal/kg/day), and a breast milk fortifier that did not contain lipids. At 3 and 6 months of corrected age, the milk type was changed according to nutritional requirements, but the same ω-6/ω-3 ratio was maintained in each group. Parents began adding complementary foods at five months of life. They kept a daily nutrition diary, and the omega-6/omega-3 ratio in foods was strictly controlled so that the two groups received similar amounts of both omega-6 and omega-3 apart from the formula. The families of all children in both groups were given the same recommendations and encouraged to introduce complementary foods together with milk. Table 1 presents the main contents of the formulas used in this study [21].

**Table 1:** Total proteins, lipids, and carbohydrate per 100 ml of formula used

Compositions of 100 ml of used formulas		Total proteins g/100 ml	Total lipids g/100 ml	Total carbohydrates g/100 ml
Group A formula (ω-6/ ω-3 = 2)	Preterm formula (16 %)	2.35	4	8.64
	First 6 months formula (13 %)	1.43	3.77	7.15
	6-12 months formula (13 %)	1.65	2.92	7.67
Group B formula (ω-6/ ω-3 = 1)	Preterm formula (16 %)	2.27	4.22	8.51
	First 6 months formula (13 %)	1.52	3.57	7.67
	6-12 months formula (13 %)	1.61	3.09	7.52
Human milk (22)	Born <29 weeks GA	2.2	4.4	7.6
	Born 32–33 weeks GA	1.9	4.8	7.5
*p* value		ns	ns	ns

[22], Bauer et al. [21]; 13 % and 16 %, the concentration of the formula (g/100 cc). Significant differences between groups (*p* <0.05, Kruskal-Wallis test)

### Methods

For fatty acid analysis, blood samples were collected from study children at 1 week of age, then at 3, 6, and 12 months. The samples were collected in tubes with anticoagulant (EDTA). After immediate centrifugation the plasma was separated and stored at −80 °C until analysis. When all samples had been received in the laboratory, plasma total fatty acids were trans-methylated as per the method described by Lepage and Roy [22]. Using tridecanoic acid as the internal standard, the fatty acid methyl esters were separated and quantified on an Agilent Technologies 7890A gas chromatograph using a flame ionization detector on a capillary column SP-2380 (Supelco, Bellefonte, PA, USA). Fatty acids were identified by comparison with commercial standards from Nu-Chek (Elysian, MN, USA) and Sigma (Madrid, Spain). Each fatty acid was quantified using electronic integration in the offline Chem Station. Nutritional content was calculated using a dietary calculation computer programme (www.odimet.es).

Anthropometric assessment was performed by the same personnel and with the same devices for both groups. For weight, a special balance was used (Seca), which had two programs: one with an expected error of 3 g and minimum and maximum limits of 40 g – 6000 g, respectively, (used until 3 months of age) and the other program with an expected error of 5 g and minimum and maximum limits of 100 g – 15 000 g, respectively, (used for infants older than 3 months). Length was measured using a special sliding infant measuring table (counter recording) with an expected error of 2 mm and a minimum and maximum of 20 cm and 100 cm, respectively (Holtain). Head circumference was determined using a non-stretchable measuring tape, measuring the perimeter at the level of the occipital prominence at the back and the mid forehead at the front.

Evoked potentials were performed using the same technique and device in both groups (Nicolet Viking IV NT), and the results were evaluated by the same trained personnel. Psychomotor development was assessed using the Brunet Lézine Scale. The Brunet Lezine scale provides an objective evaluation of a child’s maturation level in four areas: motor, coordination, language, and social [23]; an overall score ≥85 is considered normal.

### Study of other risk factors

It was important to establish if there were differences between group A and group B in terms of risk factors that could affect mental development. Therefore, sex, gestational age (assessed by prenatal echo), birth weight, APGAR, use of surfactant, sepsis, need for mechanical ventilation, use of FiO2 > 30 %, presence of intracranial haemorrhage, administration of ibuprofen for patent ductus arteriosus, and presence of bronchopulmonary dysplasia were assessed in all children who entered the study. Finally, parents were asked if they worked, and if they had a university degree. The two groups were compared, and no significant differences were found between them (Table 2).

**Table 2:** Comparison of risk factors in children between the three groups

Some risk factors	ω-6/ω-3 = 2	ω-6/ω-3 = 1	Control group	*p* value
	Group A	Group B		
	*n* = 24	*n* = 21	*n* = 25	
	n (rate)	n (rate)	n (rate)	
Male	12 (50 %)	11 (52 %)	12 (48 %)	ns
Gestation age <30 weeks	7 (29 %)	7 (33 %)	8 (32 %)	ns
Weight at birth < 1000gm	5 (21 %)	4 (19 %)	5 (20 %)	ns
APGAR at 5 min < 9	5 (21 %)	4 (19 %)	4 (25 %)	ns
APGAR at 10 min <9	2 (8 %)	2 (10 %)	3 (12 %)	ns
Need of surfactant	8 (33 %)	8 (38 %)	8 (32 %)	ns
Sepsis^a^	4 (17 %)	4 (19 %)	5 (20 %)	ns
Mechanical ventilation	9 (38 %)	6 (29 %)	8 (32 %)	ns
Need of Fio2 > 30 %	9 (38 %)	7 (33 %)	6 (24 %)	ns
Intra cranial hemorrhage (grade I or II)	3 (13 %)	3 (14 %)	4 (16 %)	ns
Need of ibuprofen	7 (29 %)	7 (33 %)	7 (28 %)	ns
Broncho pulmonary dysplasia^b^	2 (8 %)	1 (5 %)	1 (4 %)	ns
Both parents do not have job	2 (8 %)	1 (5 %)	2 (8 %)	ns
Both parents do not have university degree	13 (54 %)	12 (57 %)	12 (48 %)	ns

Data are presented as number (percentage), significant differences between groups (*p* < 0.05, Chi-square test)

^a^Preterm infants who had positive blood culture with appropriate clinical suspected status

^b^Preterm infants who had oxygen requirement either at 28 postnatal days or 36 weeks postmenstrual age

### Statistical analysis

All statistical calculations were performed with SPSS (SPSS, v.20.0, Chicago, Illinois, USA). Normality of data was analyzed with the Shapiro–Wilk test. A *p* value < 0.05 was considered statistically significant (two-tailed test), with 95 % confidence interval. Multiple testing corrections were performed using Bonferroni correction. Qualitative variables were compared between groups using a chi-square test. To evaluate Brunet Lezine score, Wilcoxon test was performed between each pair of groups (A and B, A and control, and B and control). For plasma fatty acid values and anthropometric measurements, quantitative variables were compared between groups using Wilcoxon test.

## Results

**Fig. 1:**
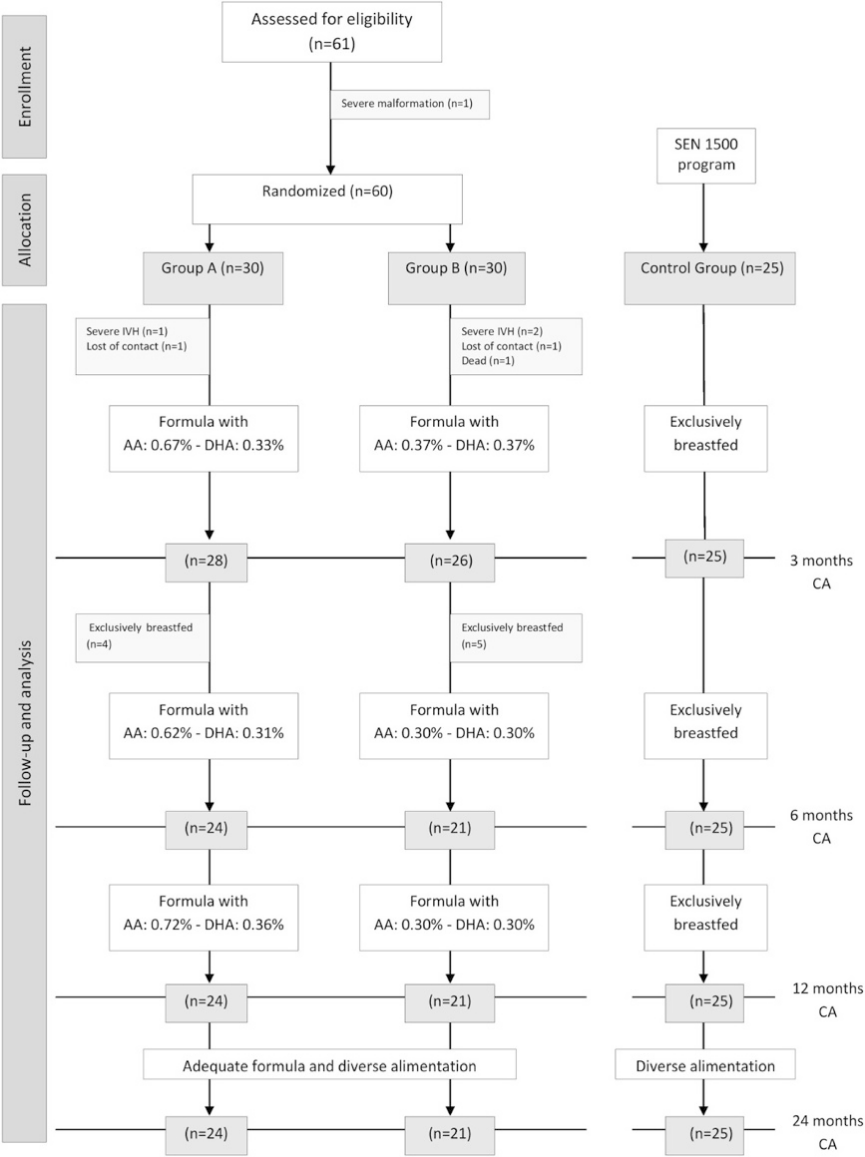
Dietary intervention and follow-up of children during the first two years of life. CA, corerected age; IVH, intraventricular hemorrhage

Sixty-one infants were recruited (Fig. 1), and 60 infants were randomly assigned to one of the two study formulas. Forty-five infants were followed up until 24 months of age.

During the first month of life, 54 of group A and 48 % of group B were fed on breast milk supported with one of the formulas. At 3 months of chronological age, 79 of group A and 76 % of group B were fed exclusively on formula, and at 6 months, 92 of group A and 86 % of group B were fed exclusively on formula. The percentage of feeds provided by breast milk in all mixed-fed infants was less than 40 % of total milk {Group A, mean 21 %, (range 10 % to 35 %). Group B, mean 26 %, (range 15 % to 40 %)}. There were no statistically significant differences between the two groups.

### Psychomotor development

At 24 months’ corrected age, the three groups were assessed using the Brunet Lézine scale. The proportion of preterm infants with a Brunet Lézine score greater than or equal to 85 was studied and compared, as was the mean score of each group, and a significant difference was noted (Table 3). Each group was then compared with the two other groups separately. There was a significant difference between group A and group B: only one child had a score less than 85 in group A, compared with six children in group B. There was also a significant difference in the average scores of groups A and B. The Brunet Lézine results of the control group, who were exclusively breast-fed, were compared with both formula groups: group A and the control group had a similar proportion of children with a score less than 85 and similar average scores. However, group B had worse results than the control group for both proportion of children with a score less than 85 and average score.

**Table 3:** Comparison of Brunet Lézine score in very preterm infants in group A, B, and control

24 months Brunet-Lézine score	ω-6/ω-3 = 2 Group A *n* = 24	ω-6/ω-3 = 1 Group B *n* = 21	*p* value	Control group *n* = 25	ω-6/ω-3 = 2 Group A *n* = 24	*p* value	Control group *n* = 25	ω-6/ω-3 = 1 Group B *n* = 21	*p* value
Mean (SD)	99.9 (9)	90.8 (11)	0.028	100.5 (7)	99.9 (9)	ns	100.5 (7)	90.8 (11)	0.007
<85	1 (4.2 %)	6 (28.6 %)	0.039	1 (4 %)	1 (4.2 %)	ns	1 (4 %)	6 (28.6 %)	0.036
≥85	23 (95.8 %)	15 (71.4 %)		24 (96 %)	23 (95.8 %)		24 (96 %)	15 (71.4 %)	

Data are presented as number (ratio) or mean (SD). Significant differences between groups (*p* <0.05, Chi-square test for comparing ratios, Wilcoxon test for comparing scores)

### Lipid profile

Some samples were not taken during follow-up due to parental refusal (one at 1 week, five at 3 and 6 months each, and three at 12 months of age). Children from group A had the following results compared with group B (Table 4): significantly higher total levels of ω-6, AA, and PUFA at 6 and 12 months corrected age; significantly higher levels of DHA and ω-3 at 6 months; and similar plasma ω-6/ω-3 ratio at 3, 6, and 12 months of age. There were no significant differences in docosapentaenoic acid (C22:5ω3), eicosapentaenoic acid (C20:5ω3), or linoleic acid (C18:2ω6) between the two groups at 6 or 12 months.

**Table 4:** Comparison of fatty acids in plasma in very preterm infants between two sample groups

Concentration in plasma (% of weight of total lipids)	Birth		3 months		6 months		12 months	
	ω-6/ω-3 = 2	ω-6/ω-3 = 1	ω-6/ω-3 = 2	ω-6/ω-3 = 1	ω-6/ω-3 = 2	ω-6/ω-3 = 1	ω-6/ω-3 = 2	ω-6/ω-3 = 1
	Group A (n = 24)	Group A (n = 20)	Group A (n = 22)	Group B (n = 18)	Group A (n = 22)	Group B (n = 18)	Group A (n = 24)	Group B (n = 18)
	Mean (SD)	Mean (SD)	Mean (SD)	Mean (SD)	Mean (SD)	Mean (SD)	Mean (SD)	Mean (SD)
Saturated fatty acids								
C12:0	1.38 (0.88)	1.02 (0.55)	2.89 (1.75)	1.17 (0.45)*	5.05 (1.85)	3.62 (1.51)	3.72 (1.08)	4.35 (1.19)
C14:0	1.40 (0.52)	1.34 (0.52)	1,90 (0.65)	1.17 (0.15)*	2.03 (0.36)	1.88 (0.35)	1.74 (0.31)	1.94 (0.38)
C15:0	0.63 (0.48)	0.48 (0.28)	0.43 (0.21)	0.49 (0.22)	0.46 (0.14)	0.42 (0.17)	0.45 (0.25)	0.37 (0.14)
C16:0	21.78 (2.17)	23.70 (3.44)	19.86 (2.78)	20.33 (1.08)	19.94 (1.05)	21.01 (0.77)	19.77 (1.00)	19.59 (0.85)
C17:0	0.51 (0.14)	0.48 (0.14)	0.46 (0.11)	0.45 (0.11)	0.35 (0.11)	0.36 (0.08)	0.38 (0.11)	0.34 (0.08)
C18:0	8.33 (1.33)	8.24 (1.43)	8.50 (1.12)	8.24 (0,79)	7.25 (0.86)	7.39 (0.70)	7.31 (0.94)	6.73 (0.89)
C20:0	0.27 (0.10)	0.23 (0.13)	0.20 (0.08)	0.28 (0.05)*	0.14 (0.05)	0.18 (0.06)	0.21 (0.04)	0.24 (0.04)
C22:0	0.90 (0.34)	1.08 (0.35)	0.70 (0.46)	0.58 (0.07)	0.60 (0.14)	0.56 (0.05)	0.55 (0.09)	0.54 (0.09)
C23:0	0.04 (0.03)	0.03 (0.01)	0.03 (0.02)	0.03 (0.03)	0.01 (0.01)	0.02 (0.01)	0.01 (0.01)	0.01 (0.00)
C24:0	0.44 (0.13)	0.40 (0.12)	0.34 (0.10)	0.38 (0.06)	0.42 (0.16)	0.39 (0.08)	0.45 (0.13)	0.39 (0.10)
C26:0	0.00 (0.00)	0.00 (0.00)	0.00 (0.00)	0.00 (0.00)	0.00 (0.01)	0.00 (0.00)	0.00 (0.00)	0.00 (0.00)
SFA	35.69 (4.24)	36.99 (4.78)	35.31 (3.49)	33.13 (1.86)	36.26 (1.59)	35.68 (1.40)	34.58 (1.25)	34.50 (1.49)
Monounsaturated fatty acids								
C14:1ω5	0.34 (0.23)	0.20 (0.07)	0.35 (0.23)	0.19 (0.17)	0.17 (0.11)	0.17 (0.09)	0.12 (0.04)	0.09 (0.01)
C16:1ω7	3.39 (1.67)	4.64 (2.48)	1.86 (2.48)	1.02 (0.41)	0.77 (0.22)	1.03 (0.40)	1.10 (0.30)	0.98 (0.34)
C18:1ω9 (t)	0.24 (0.13)	0.23 (0.11)	0.27 (0.13)	0.24 (0.05)	0.18 (0.06)	0.19 (0.05)	0.18 (0.04)	0.17 (0.05)
C18:1ω9 (c)	24.78 (2.99)	26.30 (3.36)	25.39 (2.17)	26.50 (2.63)	22.97 (1.90)	24.62 (2.02)	22.03 (2.13)	25.06 (2.52)*
C18:1ω7	0.59 (1.02)	0.49 (0.81)	0.31 (0.62)	0.14 (0.09)	0.16 (0.19)	0.36 (0.50)	0.11 (0.03)	0.14 (0.11)
C20:1ω9	0.11 (0.25)	0.05 (0.01)	0.05 (0.04)	0.07 (0.03)*	0.05 0.02)	0.05 (0.01)	0.05 (0.01)	0.05 (0.01)
C22:1ω9	0.07 (0.03)	0.08 (0.03)	0.05 (0.02)	0.06 (0.02)	0.05 (0.02)	0.05 (0.02)	0.04 (0.02)	0.05 (0.01)
C24:1ω9	1.08 (0.33)	1.26 (0.36)	0.90 (0.34)	0.96 (0.41)	0.70 (0.17)	0.87 (0.19)*	0.77 (0.18)	0.88 (0.18)
MUFA	30.60 (3.77)	33.25 (4.89)	29.18 (3.74)	29.17 (2.53)	25.04 (1.86)	27.35 (1.71)*	24.40 (1.90)	27.42 (2.53)*
Polyunsaturated fatty acids								
C18:2ω6 (t)	0.12 (0.07)	0.11 (0.05)	0.12 (0.05)	0.13 (0.02)	0.15 (0.13)	0.11 (0.04)	0.12 (0.04)	0.10 (0.01)*
C18:2ω6 (c)	19.89 (8.41)	16.57 (9.27)	23.36 (5.56)	26.43 (2.18)	26.75 (1.99)	26.91 (1.63)	28.22 (1.74)	27.68 (1.66)
C18:3ω6	1.36 (0.92)	1.42 (0.91)	0.91 (0.63)	1.36 (0.77)	1.06 (0.62)	1.64 (0.74)	1.48 (0.73)	1.69 (0.57)
C18:3ω3	0.98 (1.07)	0.84 (0.98)	0.82 (0.28)	0.95 (0.18)	0.69 (0.16)	0.84 (0.08)*	0.71 (0.17)	0.91 (0.13)*
C20:2ω6	0.10 (0.06)	0.08 (0.04)	0.09 (0.08)	0.10 (0.05)	0.08 (0.04)	0.12 (0.09)	0.08 (0.01)	0.07 (0.01)
C20:3ω9	0.10 (0.03)	0.10 (0.04)	0.09 (0.03)	0.09 (0.03)	0.06 (0.02)	0.05 (0.03)	0.00 (0.00)	0.00 (0.01)
C20:3ω6	1.45 (0.39)	1.57 (0.55)	1.14 (0.29)	1.44 (0.42)*	0.69 (0.20)	0.82 (0.27)	0.87 (0.18)	0.90 (0.18)
C20:4ω6	7.20 (1.75)	6.80 (1.57)	6.56 (1.29)	4.77 (1.09)*	6.74 (0.88)	4.22 (1.16)*	6.95 (1.55)	4.55 (0.78)*
C20:5ω3	0.16 (0.13)	0.10 (0.14)	0.12 (0.10)	0.11 (0.10)	0.06 (0.07)	0.06 (0.07)	0.02 (0.01)	0.02 (0.01)
C22:4ω6	0,37 (0.12)	0.37 (0.07)	0.29 (0.07)	0.28 (0.05)	0.28 (0.06)	0.29 (0.05)	0.38 (0.07)	0.31 (0.08)
C22:5ω6	0.05 (0.04)	0.07 (0.05)	0.07 (0.10)	0.05 (0.02)	0.04 (0.01)	0.04 (0.02)	0.04 (0.01)	0.04 (0.01)
C22:5ω3	0.05 (0.03)	0.05 (0.03)	0.04 (0.02)	0.05 (0.02)*	0.05 (0.02)	0.05 (0.02)	0.06 (0.02)	0.06 (0.02)
C22:6ω3	1.89 (0.65)	1.70 (0.47)	1.90 (0.37)	1.93 (0.25)	2.05 (0.37)	1.66 (0.29)*	2.02 (0.51)	1.67 (0.38)
Total ω6	30.54 (6.73)	26.97 (8.12)	32.55 (5.53)	34.56 (1.80)	36.76 (2.54)	34.15 (1.61)*	38.14 (2.21)	35.34 (2.43)*
Total ω3	3.07 (0.88)	2.68 (0.79)	2.87 (0.50)	3.04 (0.29)	2.84 (0.33)	2.62 (0.28)*	2.81 (0.42)	2.67 (0.39)
ω-6/ω-3	10.41 (2.91)	10.30 (2.25)	11.5 (1.9)	11.4 (1.2)	12.7 (1.7)	13.2 (1.2)	13.9 (2.7)	13.5 (2.5)
PUFA	33.71 (7.27)	29.75 (8.77)	35.51 (5.78)	37.69 (1.87)	38.69 (2.52)	36.82 (1.81)*	41.02 (2.09)	38.08 (2.32)*

Data are presented as mean (SD). *Significant differences between groups (*p* < 0.05, Wilcoxon test)

### Anthropometric measurement and evoked potentials

There were no significant differences between the two groups for the variables of weight, length, or head circumference, during the first two years of life. Likewise, there were no statistically significant differences in the results of visual- and brainstem auditory-evoked potentials at 6 and 12 months corrected age (Table 5).

**Table 5:** Comparison of somatic growth and evoked potentials between two sample groups

Comparison of somatic growth and evoked potentials between two groups		ω-6/ω-3 = 2 Group A	ω-6/ω-3 = 1 Group B	*p* value
		*n* = 24 (12 M and 12 F)	*n* = 21 (10 M and 11 F)	
		Mean (SD)	Mean (SD)	
Weight (g)	At birth M	1460 (355)	1254 (362)	ns
	At birth F	1287 (228)	1154 (176)	ns
	At 12 months M	9233 (989)	9276 (2053)	ns
	At 12 months F	8368 (1408)	7040 (711)	ns
	At 24 months M	12192 (1218)	12295 (1878)	ns
	At 24 months F	11038 (2503)	9979 (956)	ns
Length (cm)	At birth M	40.6 (3.6)	39.1 (2.8)	ns
	At birth F	38.4 (1.2)	36,4 (1.7)	ns
	At 12 months M	74.1 (2.7)	73.6 (5.8)	ns
	At 12 months F	69.8 (3.1)	68.4 (0.9)	ns
	At 24 months M	86.1 (2.7)	84.9 (4.1)	ns
	At 24 months F	82.9 (3.1)	81.7 (2,9)	ns
Head circumference (cm)	At birth M	28.4 (1.7)	27.5 (2.1)	ns
	At birth F	27.6 (1.9)	27.1 (1.8)	ns
	At 12 months M	45.7 (1)	45.9 (1.5)	ns
	At 12 months F	45.3 (1.7)	45.1 (1.1)	ns
	At 24 months M	48.6 (1)	48.4 (1.8)	ns
	At 24 months F	47.9 (1.7)	48 (0.8)	ns
		% normal	% normal	
Visual-evoked potentials	At 6 months	92 %	81 %	ns
	At 12 months	96 %	86 %	ns
Brainstem auditory-evoked potentials	At 6 months	79 %	86 %	ns
	At 12 months	83 %	90 %	ns

*cm* centimeter, *g* gram, *M* male, *F* female, ns indicating no significant differences between groups (*p* < 0.05, Wilcoxon test for growth, Chi-square test for potentials)

## Discussion and conclusions

This trial studied very preterm infants who received supplementary formulas with different AA/DHA ratios (group A, 2/1 and group B, 1/1). Group A had significantly higher plasma values of arachidonic acid and polyunsaturated fatty acids than group B. Group A also achieved a higher average Brunet Lézine score at 24 months than group B.

Regarding Brunet Lézine score at 24 months, there was a statistically significant difference between the two groups: in group A (ω-6/ω-3 = 2/1), only 4.2 % had a score less than 85. Also, group A had a higher mean score than group B. This could have been due to the higher quantity of ω-6 or PUFAs that group A were fed, as well as the higher plasma levels of ω-6 and PUFAs in group A compared with group B. To explain these results, it must be remembered that AA is a key component of cell membranes, serves as a precursor to prostaglandin formation, and is clearly involved in the signalling systems of the brain [15]. Whilst it is acknowledged that maternal milk is the ideal milk and generally contains a higher concentration of AA than formula milk [8, 28, 29], the amount of AA needed by preterm infants to maintain ideal brain development is not clear, and more studies and further discussion are required. To clarify the results of this study, we reiterate that the two formula groups were randomly assigned, then observed and followed closely during the first two years of life. From the beginning of the study, the two groups had similar gestational ages, weights at birth, APGAR at 5 and 10 min, and incidence of other risk factors that could affect neurodevelopment (e.g., sepsis, intracranial haemorrhage, bronchopulmonary dysplasia). The study aimed to provide similar nutrition to all preterm infants in the two groups during this period. The two study groups were also compared to a breast milk-only group. However, the two study formulas differed not only in the proportion of omega 6 and omega 3 LCPUFAs, but also in the total amount of LCPUFAs. It cannot be excluded that the better performance of infants fed with formula A could be attributable to the total quantity (and not the ratio) of LCPUFAs.

International groups have published recommendations for the optimal levels of ω-3 and ω-6 during the first three years of life, with recommended ω-3 supplementation ranging from 0.6 % to 2 % of daily energy requirements compared with 5 %-10 % of ω-6 (almost a 10-fold difference) [24] The European Society for Paediatric Gastroenterology, Hepatology and Nutrition (ESPGHAN) Committee on Nutrition suggests that the daily intake for a preterm infant include the following: DHA, 12 to 30 mg/kg (≈0.2 %-0.5 % of all fatty acids) and AA, 18 to 42 mg/kg (≈0.3 %-0.7 % of all fatty acids) [25].

Multiple studies have confirmed the importance of PUFAs in preterm infants’ mental development and have reported that preterm infants fed with supplemental PUFAs (especially DHA) showed improved brain development. However, none of these trials clearly recommended an ω-6 to ω-3 ratio [11, 12, 26].

A review of the scientific literature showed that the AA/DHA ratio in formula milk (often 1/1) is different from the AA/DHA ratio in breast milk. In a review of 65 studies involving 2474 women, the mean concentration of DHA in breast milk was 0.32 % (range: 0.06 %–1.4 %) and that of AA was 0.47 % (range: 0.24 % –1.0 %) [27]. In a study on FA composition of mothers’ milk at 3, 7, and 28 days post-partum in term and preterm infants, the ratio of AA/DHA was approximately 3/1 and 2/1, respectively, with DHA levels between 0.5 %-1.0 %, and AA levels between 1.3 %-2.6 % [28]. Also, in one investigation conducted in nine countries concerning breast milk for term neonates, 7 countries reported an AA/DHA ratio greater than 1/1 and only 2 countries reported a ratio of less than 1/1 (range, from 0.51/1 in Japan to 3.16/1 in the USA) [29]. In a new study of term infants in the USA, the AA/DHA ratio in breast milk was 2.64/1 at birth and 2.81/1 at 6 weeks [8].

Interestingly, despite having different formula ratios of AA/DHA, the same quantity of DHA, and almost the same proportion of mixed-fed children (breast-fed with formula) in groups A and B, there was almost no difference between the two groups in plasma ω-6/ω-3 ratio and group A had higher DHA levels than group B at 6 months. This could have been because α-Linolenic acid and Linoleic acid compete for the same enzyme pathways to form DHA and AA, respectively [30], and since group A had higher AA supplementation, the internal balance of LCPUFA synthesis could have shifted towards DHA. In addition, plasma omega-6/omega-3 ratios were not expected to be similar, and this result could have been because omega-6 and omega-3 have many components besides AA and DHA. The balance of omega-6 and omega-3 is highly complex, and our trial is the first in addressing AA, as previous trials have focused on DHA and overlooked AA. Therefore, further studies are required to understand the complex relationship between omega-6 and omega-3. However, plasma PUFAs were higher in group A than in group B. This means that the increased quantity of AA in the milk given did not affect the balance between ω-6 and ω-3. This balance is considered very important in human health and growth [9].

A recent review showed that LC-PUFA supplementation during pregnancy was associated with modest increases in birth size in both low-income and high-income populations. However, postnatal supplementation with LC-PUFAs did not influence infant growth [31]. Throughout our study, anthropometric growth was measured during the first two years of life in groups A and B, and weight, height, and head circumference growth were similar in the two groups. Therefore, these results suggest that higher formula levels of omega-6 do not affect anthropometric growth.

The VEP results of group A and group B were compared, showing no major differences in visual function, a result that supports the unique importance of DHA in visual pathway maturation in preterm infants. Underscoring this, DHA levels have been shown to reach 30 %-40 % of total fatty acids in rod photoreceptor outer segments of the human retina [32] and the two formula groups received the same quantity of DHA. This result was despite of the different plasma DHA values at 6 months.

Theoretically, the auditory tracts are neural fibres, and LC-FUFAs are thought to be important in their development and maturation. However, no studies were found to support this hypothesis. Paradoxically, one study showed no effect of LC-PUFA on auditory brain-stem evoked responses [33]. Due to the above data, no important differences were anticipated between the two groups in terms of auditory function, despite one group having double the quantity of AA.

All mothers were from the same geographical area, were of the same ethnicity and religion, and had similar dietary habits. There are several studies on the omega-6/omega-3 ratio in breast milk confirming minimal differences in this ratio in the same geographical region, and marked differences only between distant parts of the world, because of distinct dietary habits [27, 29]. Therefore, in this study, the breast milk omega-6/omega-3 ratio was not measured, as it was understood that it would contain a similar ratio, and that the significant difference in the omega-6/omega-3 ratio was between the supplemental formulas only. Also, the number of mixed-fed babies decreased significantly after the first month of life (from the first month to the third month, from 54 % to 21 % in group A and from 48 % to 24 % in group B).

There was a variety of factors that were difficult to control, for example the socio-economic status and the educational level of the mother [34, 35]. These factors could have played an important role in neurodevelopment and cognitive function. Finally, the relatively small sample sizes (as a result of more children than expected being lost to follow-up) could be another limitation of this trial.

In conclusion, the improved LCPUFA plasma concentrations and psychomotor development in very preterm infants despite the same formula quantity of DHA could have been due to the double mount ratio of AA to DHA or the higher total quantity of LCPUFAs. Nevertheless, further studies are required to support the benefits of a balanced AA/DHA ratio (more AA) in preterm infant supplementation.

## Abbreviations

AA: Arachidonic acid
DHA: Docosahexaenoic acid
GA: Gestational Age
LCPUFA: Long chain polyunsaturated Fatty Acids
VLBW: Very low birth weight
ω-3: Omega 3
ω-6: Omega 6
